# Molecular detection and pathogenicity of a nucleopolyhedrovirus isolated from looper caterpillar (*Hyposidra talaca*), a tea pest

**DOI:** 10.1007/s13205-016-0568-6

**Published:** 2016-11-16

**Authors:** Soma Dasgupta, Hijam Ranjit Singh, Sudripta Das, Sunil Kumar Pathak, Rakesh Kumar Bhola

**Affiliations:** 1 Plant Protection Department, Tea Research Association, North Bengal Regional Research and Development Centre, Nagrakata, Jalpaiguri, 735225 West Bengal India; 2 Department of Biotechnology, Tea Research Association, Tocklai Tea Research Institute, Jorhat, Assam 785008 India; 3 Advisory Services of Assam, Tea Research Association, Tocklai Tea Research Institute, Jorhat, Assam 785008 India; 4 Department of Zoology, Gauhati University, Guwahati, 781014 India; 5 Department of Medicinal Plant, Institute of Bioresources and Sustainable Development (IBSD), Imphal, Manipur 795004 India

**Keywords:** *Hyposidra talaca*, Nucleopolyhedrovirus, Polyhedrin gene, PCR, Bioactivity

## Abstract

*Hyposidra talaca* is a major defoliating pest of tea plants in north-eastern part of India. In this study, we look for variations (if any) in the attacking virus. Viral samples were collected from different regions of the northern part of West Bengal in India and were analyzed by PCR technique to study the variations across the region. The partial segment of the HytaNPV polyhedrin gene was cloned and sequenced. A 527 bp nucleotide sequence containing highly conserved region from polyhedrin gene of HytaNPV was observed. The blast homology search for studied polyhedrin gene showed 98% sequence identity with the sequence of previous reported NPV of *H. talaca*, *H. infixaria* and *Buzura suppressaria.* Pathogenicity study against second instar *H. talaca* indicated that the LC_50_ values ranged from 4.61 × 10^5^ to 7.57 × 10^5^ polyhedral occlusion bodies per ml (POBs/ml) and the LT_50_ values ranged from 4.2 to 6.66 days. Sequencing result reveals that the same HytaNPV strain dominates across this area and the pathogenicity indicates its potential as an alternative to chemical insecticides to control *H. talaca.*

## Introduction

Baculoviruses are renowned for most widely studied virus of insects. More than 700 baculoviruses have been isolated mainly from the insect species of the orders Diptera, Hymenoptera and Lepidoptera. Nucleopolyhedroviruses (NPVs), which are a part of baculovirus, are considered as potential biocontrol agents and have been applied successfully against larvae of many insect pests in the world (Hu et al. 1993; Ma et al. 2007). NPVs are mainly pathogenic for insects of Lepidoptera. Polyhedrin gene of NPV is considered as one of the most conserved baculovirus genes (Jehle 2004), and for the development of the genetic amplification technique, this gene proved its efficiency (Woo 2001). Polymerase chain reaction (PCR) technique can amplify target DNA sequence and it has been used in baculovirus screening since the last three decades (Burand et al. 1992; Ikuno et al. 2004; Galal 2009; Hewson et al. 2011; Arneodo et al. 2012). The PCR technique is suitable for diagnosis of viruses as identification, characterization as well as for their genome investigation (Moraes and Maruniak 1997; Fattouch et al. 2005).

The characteristics of NPVs that empower it to be useful as biopesticide are its high pathogenicity, host specificity, less chances of cross-infectivity and having the ability of rapid distribution. The virus has stable occlusion bodies and can effectively suppress a target pest from a crop field (Moscardi 1999). In this regard, *Hyposidra talaca* nucleopolyhedrovirus (HytaNPV) is an extremely infectious natural agent causing most destructive disease to the tea pest *Hyposidra talaca*. In India, HytaNPV is reported in Terai and Dooars part of North Bengal (Mukhopadhyay et al. 2010; Antony et al. 2011; Sinu et al. 2015). The development of such control agent starts with the isolation of naturally occurring entomopathogens and followed by their identification, characterization as well as the evaluation of their virulence.

The *H. talaca* looper caterpillar (Walk.) (Lepidoptera: Geometridae) is an economically significant defoliating pest of the tea belt in the north-eastern part of India. Previously, *Buzura suppressaria* (Guen.) (Lepidoptera: Geometridae), a major tea defoliator in India and China, was dominant in this region, but gradually it was suppressed by the current destructive pest *H. talaca* (Hazarika et al. 2009). In the last decade, *H. talaca* was introduced into tea agro-ecosystem and caused severe damages in the tea industry. It spread all over the northern part of West Bengal (WB) and Assam in 2006 (Sinu et al. 2011). As the management of *H. talaca* is carried out with their population monitoring and respective damage control application of chemical pesticides, which are mainly organophosphates and synthetic pyrethroids (Sannigrahi and Talukdar 2003; Sarker and Mukhopadhyay 2006), there is an urgent need to find an alternative control method. Among few natural enemies of the pest, HytaNPV is found to be active and causes epizootics within a dense population of *H. talaca* (Sinu et al. 2015). Successive generations of insect host are infected by polyhedra; thus the host serves as a reservoir of inoculums.

Due to the high potency of the virus as chemical alternative, finding out the naturally improved isolates of the virus is a matter of great interest. PCR-based diagnosis was used for identification, establishment of relationship and also to find out the variation within the tested HytaNPVs. The primary aim of the study was to identify the most potential isolate of HytaNPV. Examination of the samples from four different geographic locations was done for the above-mentioned identification and simultaneously infectivity evaluation was conducted to determine their successful practical use, by laboratory bioassay. This study may play an important role in the programs of integrated pest management (IPM).

## Materials and methods

### Larval rearing

To investigate the sensitivity of *H. talaca*, the second instar larvae of this looper caterpillar were originally collected from Dooars region, West Bengal, India. The collection site was free of pesticides since 6 months and the larvae were carried with leaves to reduce their transportation disturbances. They were reared in the laboratory of the Plant Protection Department, North Bengal Regional Research and Development Centre, Tea Research Association (TRA), for three successive generations at 28 ± 2 °C, 72 ± 3% relative humidity and a 13L:11D photoperiod. Fresh tea leaves were provided as food after sterilizing with 10% formalin for 5 min and rinsed with sterile double-distilled water. These larvae were used for viral propagation and bioassays. The larvae were also examined daily to eliminate the secondary infection.

### Viruses

The HytaNPV samples, used in the PCR study, were collected during 2012–2013 from geographically distinct localities covering eastern Dooars, western Dooars, central Dooars and Terai region of West Bengal, India. The average rainfall of Terai and Dooars are approximately 3500 and 3160 mm, respectively, situated between 26° 16′ and 27° 12′ north latitudes and 87° 59′ and 89° 53′ east longitudes. The virus samples were collected from ten tea estates located across these four distinct geographic regions (Table 1). From each collection site, 50–60 virus-infected larvae were sampled. The virus-infected larvae were collected as samples considering the primary signs of typical NPV infection in larval stage like flaccidity, rupturing of the cuticle and hanging upside down by their abdominal legs. Later, the presence of HytaNPV was ascertained by microscopic view and PCR study. The individual larva was collected in 1.5 ml centrifuge tube and the collected samples were kept at −20 °C for future use.

**Table 1:** Details of the survey site location

Region	Survey site	Isolate referred as	Geographic coordinate
Western dooars	Kumlai tea estate	KML	26°50′N, 88°41′E
	Washabari tea estate	WSB	26°52′N, 88°32′E
Central dooars	Bhagatpur tea estate	BGP	26°53′N, 88°55′E
	Ambari tea estate	AMB	26°52′N, 89°03′E
	Karbala tea estate	KRB	26°47′N, 89°04′E
	Telipara tea estate	TLP	26°43′N, 89°03′E
	Bara Dighi tea estate	BRD	26°47′N, 88°46′E
	Lakhipara tea estate	LKP	26°49′N, 89°00′E
Eastern dooars	Rajabhat tea estate	RJB	26°39′N, 89°29′E
Terai	Simulbari tea estate	SMB	26°47′N, 88°18′E

### Purification of HytaNPV

For molecular analysis, each of aqueous homogenate of viral samples containing polyhedral occlusion bodies (POBs) was purified by two rounds of centrifugation. The putrefied samples were homogenized with pestle and mortar, and the concentrates were diluted with sterile double-distilled water. The crude filtrate was initially centrifuged for 5 min at 600 rpm to remove larger contaminants. The supernatant was collected in a new centrifuge tube for further centrifugation at 8000 rpm for 20 min to pellet the virus and then washed with sterile double-distilled water for three times. The pellets were finally dissolved in 1 ml sterile double-distilled water and conserved at −20 °C. The quantification of POBs was carried out by using Neubauer hemocytometer (Marienfeld, Germany) under phase-contrast microscope (Olympus BX 51). For bioassays, the viral samples were purified by following the same method.

### Virus propagation

Four HytaNPV isolates (SMB, RJB, WSB and TLP), belonging to four different geographic regions, were taken for bioassay tests and multiplied in second instar *H. talaca* larvae for mass propagation. In case of each isolate, the multiplication of HytaNPV was conducted by isolation of POBs from infected cadavers. For multiplication of each isolate, 150 larvae were fed on tea leaves dipped with virus suspension in a concentration of 10^7^ POBs/ml. After 2 h of starvation, they were exposed to virus-contaminated leaves for 24 h. Virus-induced larvae were kept as 30 larvae per group and reared in the laboratory at 28 ± 2 °C, 72 ± 3% relative humidity and a 13L:11D photoperiod. Microscopic view confirmed the presence of POBs in the cadavers of virus-induced larvae.

### Viral DNA extraction and PCR amplification

After following the purification method, viral DNA was extracted from POBs of each of ten field collected HytaNPV samples and purified with QIAamp DNA Mini Kit (Qiagen) according to the manufacturer’s protocol. The final DNA was obtained after cleaning several times with ethanol and diluted wash solution. Later, DNA was eluted and re-suspended in 20 µl molecular-grade water (Himedia). The isolation of DNA was confirmed by electrophoresis in 1% agarose gel and quantified with BioPhotometer (Eppendorf).

A highly conserved region of polyhedrin gene from HytaNPV isolates was amplified. The PCR was performed using the degenerate primer (F: 5′-GGACCSGGYAARAAYCAA AAA-3′ and R: 5′-GCRTCWGGYGCAAAYTCYTT-3′) designed according to Antony et al. (2011). The PCR reaction was carried out taking 50–100 ng of viral DNA in a 25 µl reaction solution containing 1X PCR buffer (Invitrogen, USA), 1.5 mM MgCl_2_ (Invitrogen, USA), 0.5 mM dNTPs (Bangalore Genei, India), 1 U Platinum *Taq* DNA polymerase (Invitrogen, USA) and 0.5 µM of each primer. PCR amplification was performed in a DNA thermal cycler (Veriti Thermal Cycler, Applied Biosystems, CA, USA). The conditions used were initially denaturation for one cycle at 94 °C for 5 min, followed by 35 repeated cycles of 94 °C for 30 s, 50 °C for 30 s, 72 °C for 30 s, and the final extension cycle at 72 °C for 7 min. The amplified products were resolved in 1.5% agarose gel stained with ethidium bromide.

### Cloning and sequencing of the polyhedrin gene

The positive PCR product of the SMB isolate was eluted from the gel using HiPura Agarose Gel DNA Purification Spin Kit (Himedia, India) following the manufacturer’s protocol. The eluent was ligated into the pGEM-T vector (Promega, UK) in a 3:1 (insert: vector) molar ratio with T_4_ DNA ligase as described by the manufacturer. The ligated products were transformed into *Escherichia coli* DH10β competent cells (Invitrogen, USA). Following amplification of recombinant clone with M13 universal forward and reverse primers, the sequencing was performed using the BigDye Terminator v1.0 cycle sequencing kit (Applied Biosystems) in ABI 3500 Genetic Analyzer (Applied Biosystems).

### Sequence comparison and phylogenetic analysis

The sequence data were assembled into contig by DNA Dragon Version 1.5.6 (SequentiX, Germany) followed by the multiple-sequence alignments construction with the highly similar DNA and protein sequences using the Clustal W program (Thompson et al. 1994). The obtained nucleotide sequence was blasted to the GenBank database (blastx) to retrieve similar nucleotide and amino acid sequences, which were used for phylogenetic tree construction. The phylogenetic tree of aligned amino acid sequences was generated by the neighbor-joining (NJ) algorithm (Saitou and Nei 1987) using Molecular Evolutionary Genetics Analysis (MEGA) v6.06 (Tamura et al. 2013). To estimate the confidence limits of nodes, 1000 bootstrap samples were generated. To reduce the impact of partial sequences of polyhedrin available in the GenBank database and to maximize the use of the available information, the pairwise comparison of amino acid sequences was performed by ClustalW with the default parameters.

### Bioassays

The biological activity of four HytaNPV isolates, viz., SMB, RJB, WSB and TLP (belonging to four different geographic regions), was tested against early second instar *H. talaca* larvae. Bioassay tests were conducted using purified viral suspension by leaf-dip feeding technique. The virus concentrations were quantified with a phase-contrast microscope and a Neubauer hemocytometer. The concentrations of each tested isolates were prepared from the following stock concentrations in POBs/ml: 3.6 × 10^9^, 5.7 × 10^9^, 2.9 × 10^9^ and 8.1 × 10^9^ for SMB, RJB, WSB and TLP isolates, respectively. The experiments were performed using six concentrations of each virus isolate containing 1 × 10^3^, 1 × 10^4^, 1 × 10^5^, 1 × 10^6^ 1 × 10^7^ and 1 × 10^8^ POBs/ml. Larvae were taken from the virus-free rearing culture and starved for 2 h before feeding viruses. Leaves were dipped in viral suspension, air-dried and fed to larvae for 24 h prior to feeding on fresh foliages until death or pupation. In the control treatment, the virus suspension was replaced by double-distilled water. For each isolate, three replicates each of 30 larvae were used for each virus concentration and three replicates (30 larvae per replication) of double-distilled water-treated leaves served as control. Larvae were incubated at 27 ± 1 °C, 72 ± 3% RH and a 13L:11D photoperiod. Virus-induced cumulative mortality was recorded daily till death and the mortality response data were analyzed on the basis of mortality on day 7 post-inoculation. The diseased cadavers were collected and kept in −20 °C for further analysis. Later virus infections were confirmed by the presence of POBs in the cadavers, when viewed under phase-contrast microscopy.

### Statistical analysis

The mortality of larvae was tabulated daily for all NPV isolates vs. dose combinations (4 × 6). Probit analysis was performed using IBM SPSS release 23.0.0.0 on the basis of mortality data obtained after 7 days of post-inoculation. The median lethal concentrations (LC_50_) for second instar were obtained from the SPSS probit model. Median lethal time (LT_50_) was also determined for each concentration using the equation (Biever and Hostetter 1971):

$${\text{LT}}_{50} = a + e(c - b)/D$$

where *‘a’* is the number of hours from the initiation of the test until the reading made immediately before the 50% mortality was recorded; *‘b’* is the total number of larvae dead at the reading immediately before the 50% mortality was recorded; *‘c’* is the 50% of the total number tested (in our case, it is 45); *‘D’* is the number of larvae dying in 24 h during which the 50% mortality was reached; and *‘e’* is the number of hours between mortality counts (24 h in this case).

## Results

### Field survey

**Fig. 1:**
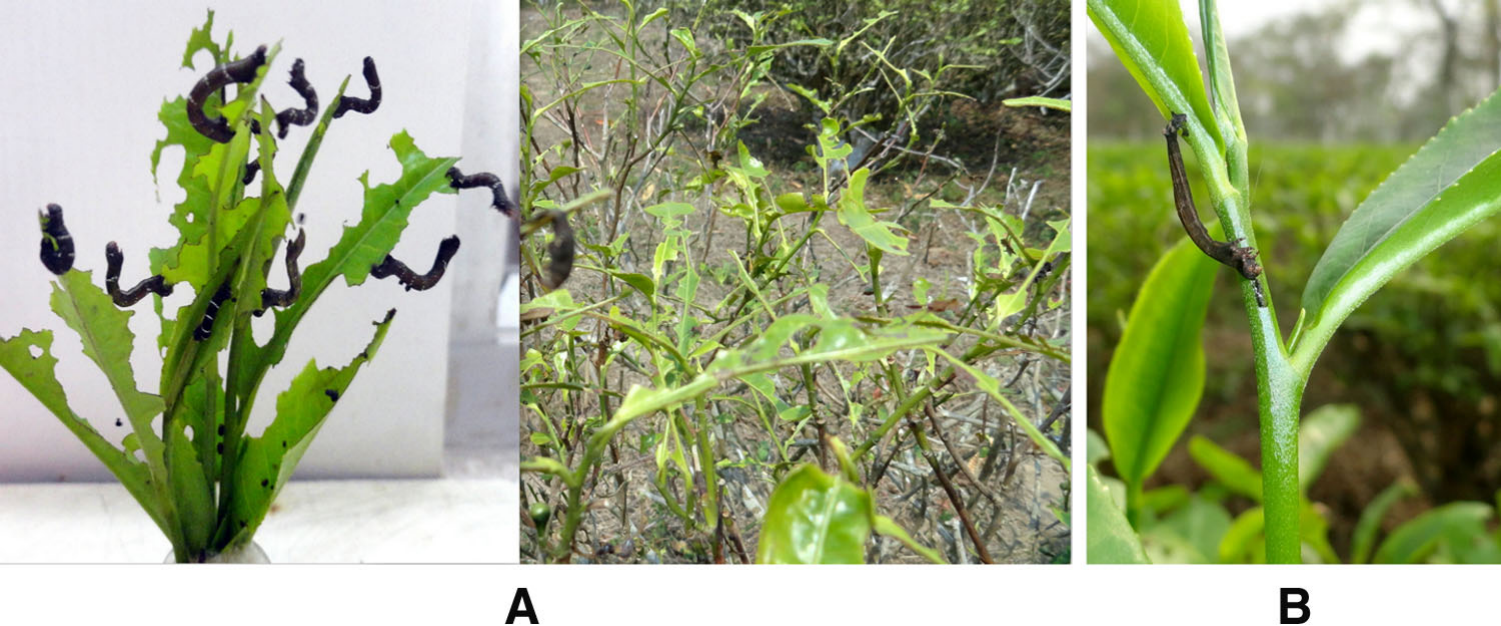
**a** Tea leaves affected by *Hyposidra talaca.* **b** Cadaver of *Hyposidra talaca* showing symptoms of nucleopolyhedrovirus infection

The virus-infected larvae were collected from different geographic locations (Dooars and Terai) by primary observation of typical NPV signs and subjected to further studies (Fig. 1). The virus was diagnosed by microscopic study, propagated in *H. talaca* laboratory colony, purified and kept at −20 °C for future studies. The number of polyhedral occlusion bodies was calculated by a hemocytometer showing the presence of approximately 8.7 × 10^9^ POBs/ml of cadaver.

### PCR amplification and sequencing

**Fig. 2:**
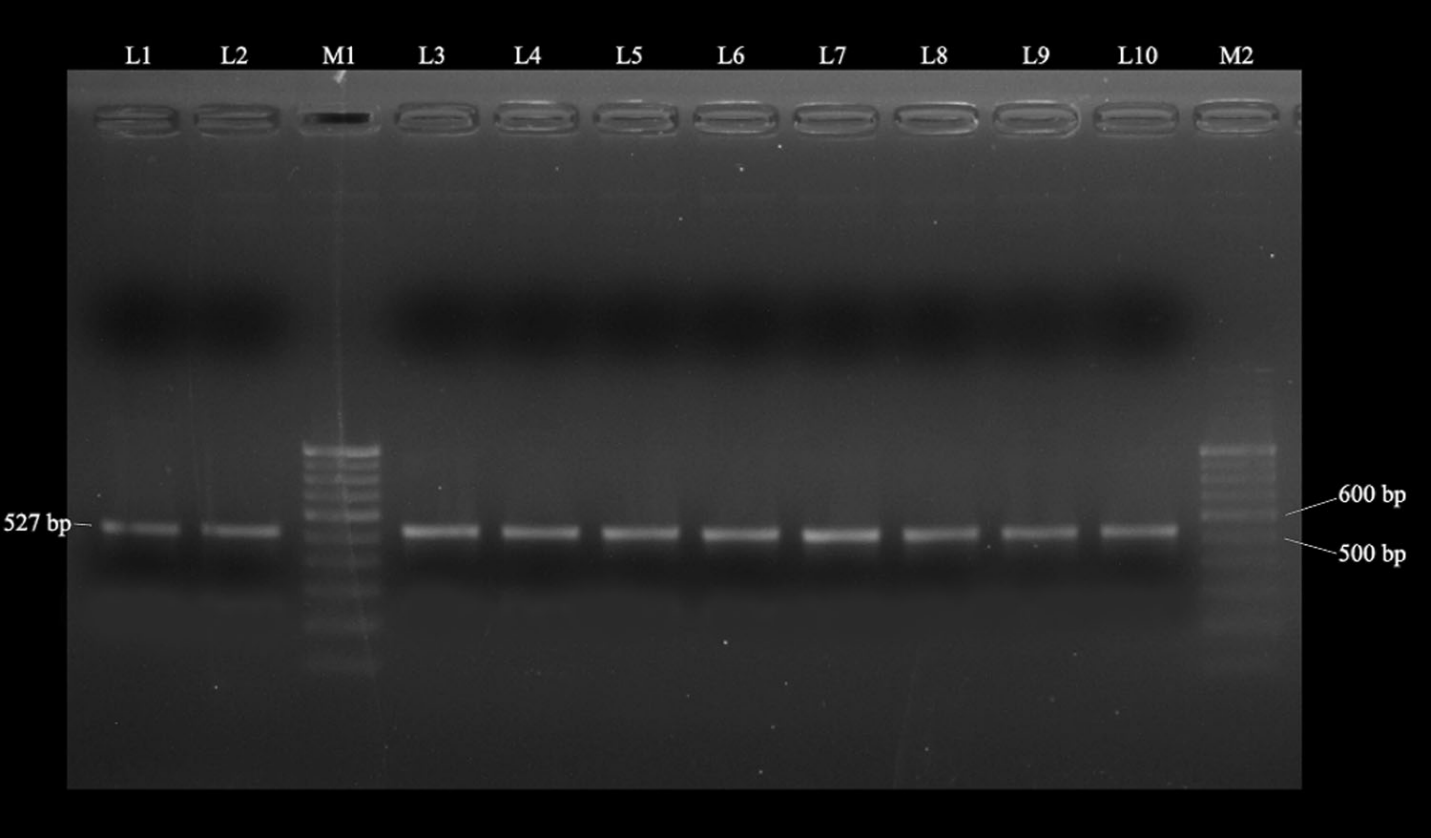
Agarose gel electrophoresis of specific PCR-amplified products from ten HytaNPV isolates. Lanes M 1 and M 2, 100 bp Ladder DNA marker (Genei, Bangalore); *Lane L1*: SMB, *L2*: LKP, *L3*: KRB, *L*4: TLP, *L5*: KML, *L6*: WSB, *L7*: BRD, *L*8: RJB, *L9*: BGP and *L10*: AMB isolate, respectively. The size of the band is shown in bp (base pair)

The degenerate primer set was used to amplify the targeted sequence from the isolated DNA of the purified polyhedra and successful amplification of the specific polyhedrin gene region of ten isolates of HytaNPV was performed. Amplification was not obtained from the extract of control larvae. The bands obtained from the PCR products of all ten isolates were identical (Fig. 2). Hence, the sequencing was performed by taking only the SMB isolate of the Terai region; then it was compared and cross validated with previously reported HytaNPV (from Dooars region). Hereafter, the studied Terai isolate is denoted as HytaNPV-P. The amplified product of the concerned isolate showed a nucleotide sequence of 527 bp (GenBank accession no. KP027542). The deduced protein consists of 175 amino acids with the predicted molecular mass of 19.52 kDa.

**Fig. 3:**
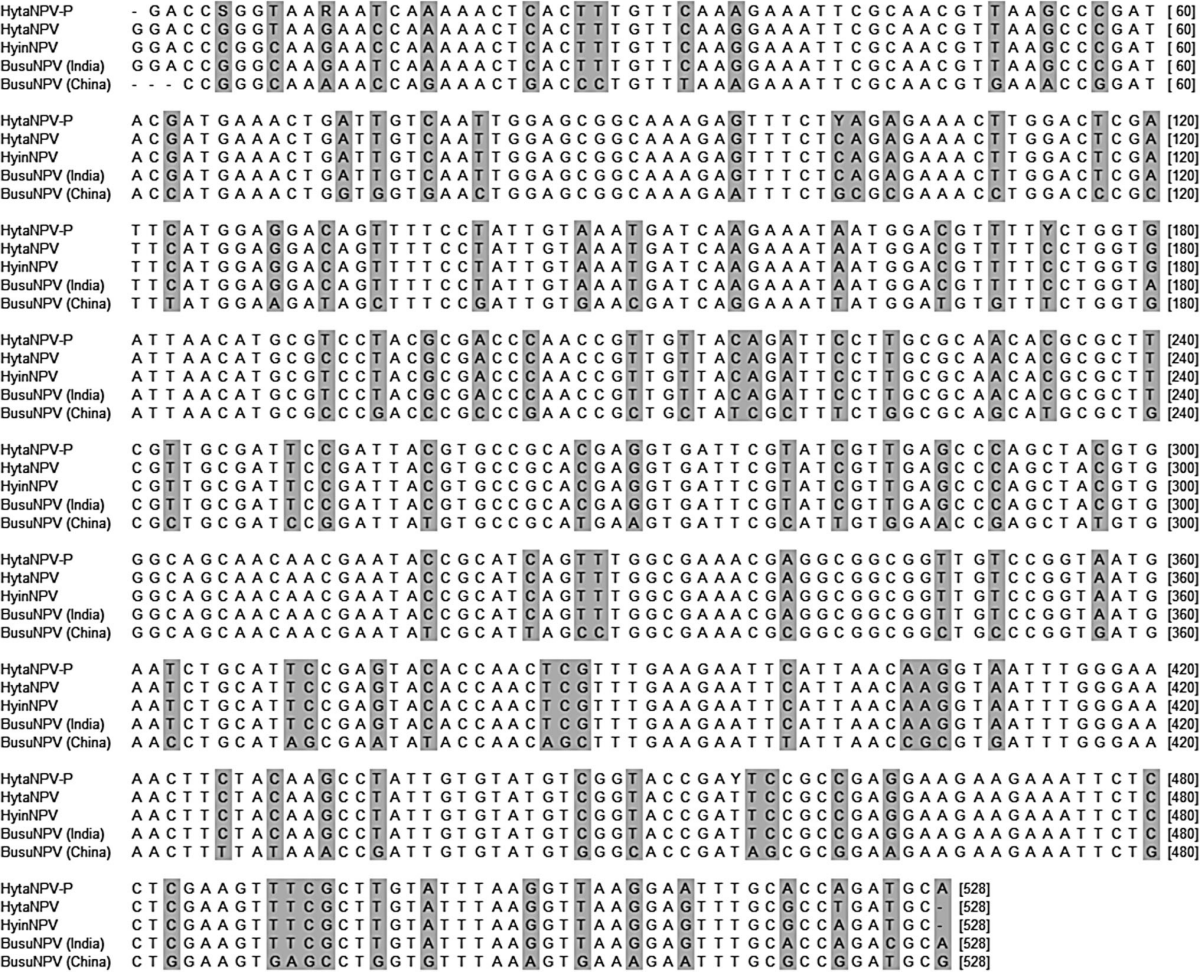
Comparison of HytaNVP-P polyhedrin gene sequence (acc. no. KP027542) with the sequences of HytaNPV, HyinNPV, BusuNPV (Indian isolate) and BusuNPV (Chinese isolate). Positions containing different nucleotides are *shaded*

The obtained nucleotide sequence was blasted in the NCBI GenBank database, the NPV polyhedrin gene was identified and the blastn algorithm indicated the significant homology with 29 NPV isolates. The blast results for the polyhedrin gene of HytaNPV-P (acc. no. KP027542) at the nucleotide level revealed the highest homology of 98% with the polyhedrin gene of HytaNPV (acc. no. JF510035)*, H. infixaria* NPV (HyinNPV, acc. no. JF510036) and *B. suppressaria* NPV (BusuNPV, acc. no. JF510034) previously reported by Antony et al. (2011) and the lowest sequence identity of 79% was observed in *Plusia orichalcea* NPV (acc. no. AF019882). On comparing, the multiple-sequence alignment of nucleotide was generated with HytaNPV-P, HytaNPV, HyinNPV and BusuNPV of Indian and Chinese isolate (Fig. 3). In comparison with previously reported HytaNPV, it exhibited ten nucleotides changes (at 6, 12, 15, 36, 102, 174, 192, 513, 519 and 522 positions) throughout the DNA segment.

**Fig. 4:**
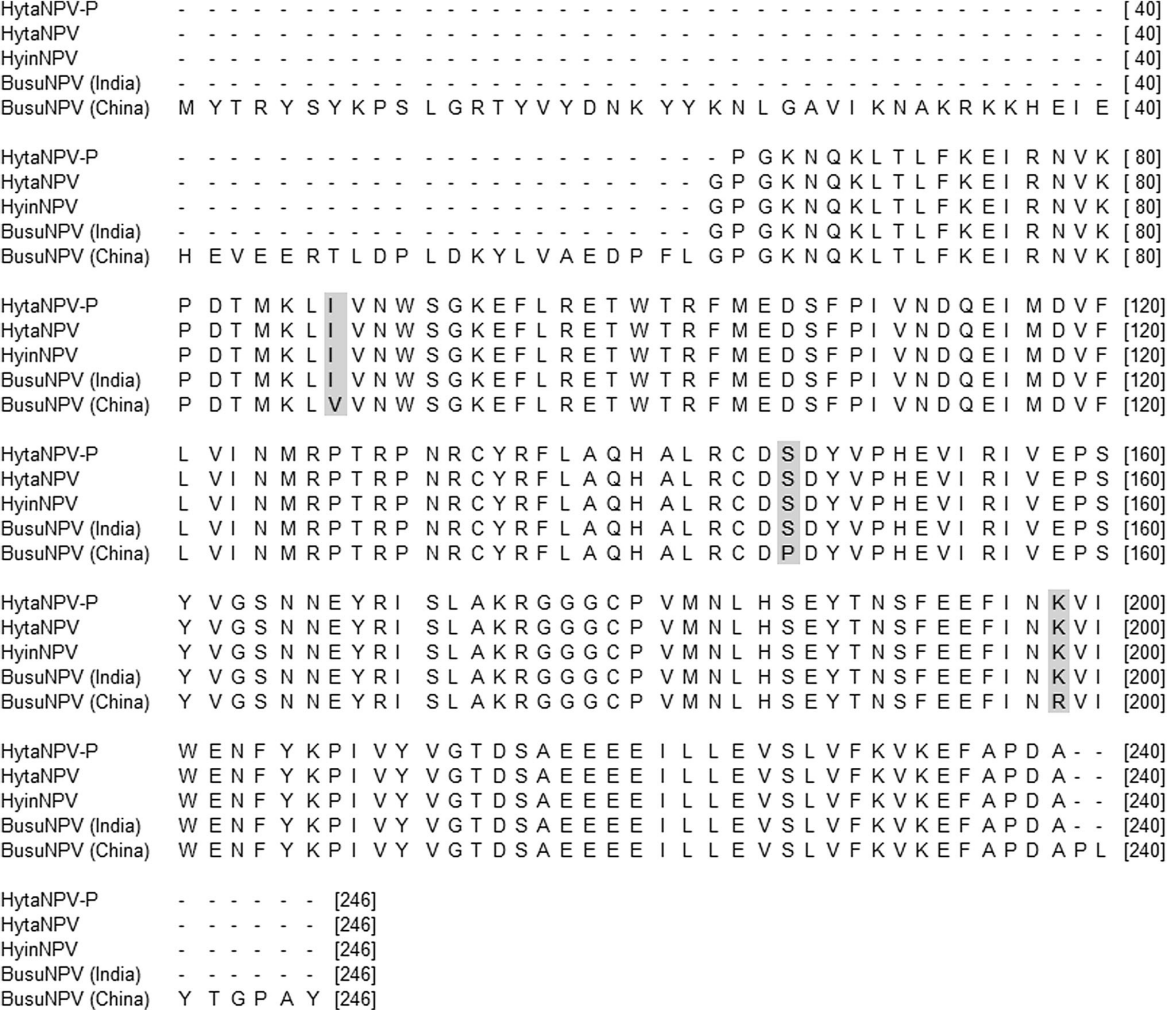
Deduced amino acid sequence alignment of HytaNPV-P poyhedrin gene (acc. no. AJN00735) with HytaNPV (acc. no. AEK86285), HyinNPV (acc. no. AEK86286), BusuNPV (Indian isolate, acc. no. AEK86284) and BusuNPV (Chinese isolate, acc. no. YP009001778). Positions containing different amino acids are *shaded*

The deduced amino acid sequence was compared to all other reported polyhedrins in the GenBank database, and HytaNPV-P polyhedrin (acc. no. AJN00735) showed 87–100% homology with other lepidopteran NPVs. The results showed 100% sequence homology with HytaNPV (acc. no. AEK86285), HyinNPV(acc. no. AEK86286) and BusuNPV (acc. no. AEK86284). In spite of the above similarities, it created 98% sequence identity with the Chinese isolate of BusuNPV (acc. no. YP009001778), 97% sequence identity with *Ectropis obliqua* NPV (EcobNPV, acc. no. YP874194; also reported from China), and *Hemileuca* sp. NPV (acc. no. YP008378217), and 95% sequence identity with *Helicoverpa armigera* NPV (acc. no. ABW06597) and *Mamestra configurata* NPV (acc. no. NP613084). The lowest sequence identity of 88% was observed with *Bombyx mori* polyhedrin (acc. no. NP047414). The multiple-sequence alignment for amino acid was also performed taking the same NPV isolates as described in nucleotide alignment (Fig. 4). There was no difference found with previously reported HytaNPV, HyinNPV and BusuNPV (Indian Isolate). A difference of three amino acids (at 87, 146 and 198 positions) was noticed with the BusuNPV of the Chinese isolate.

### Phylogenetic analysis

**Fig. 5:**
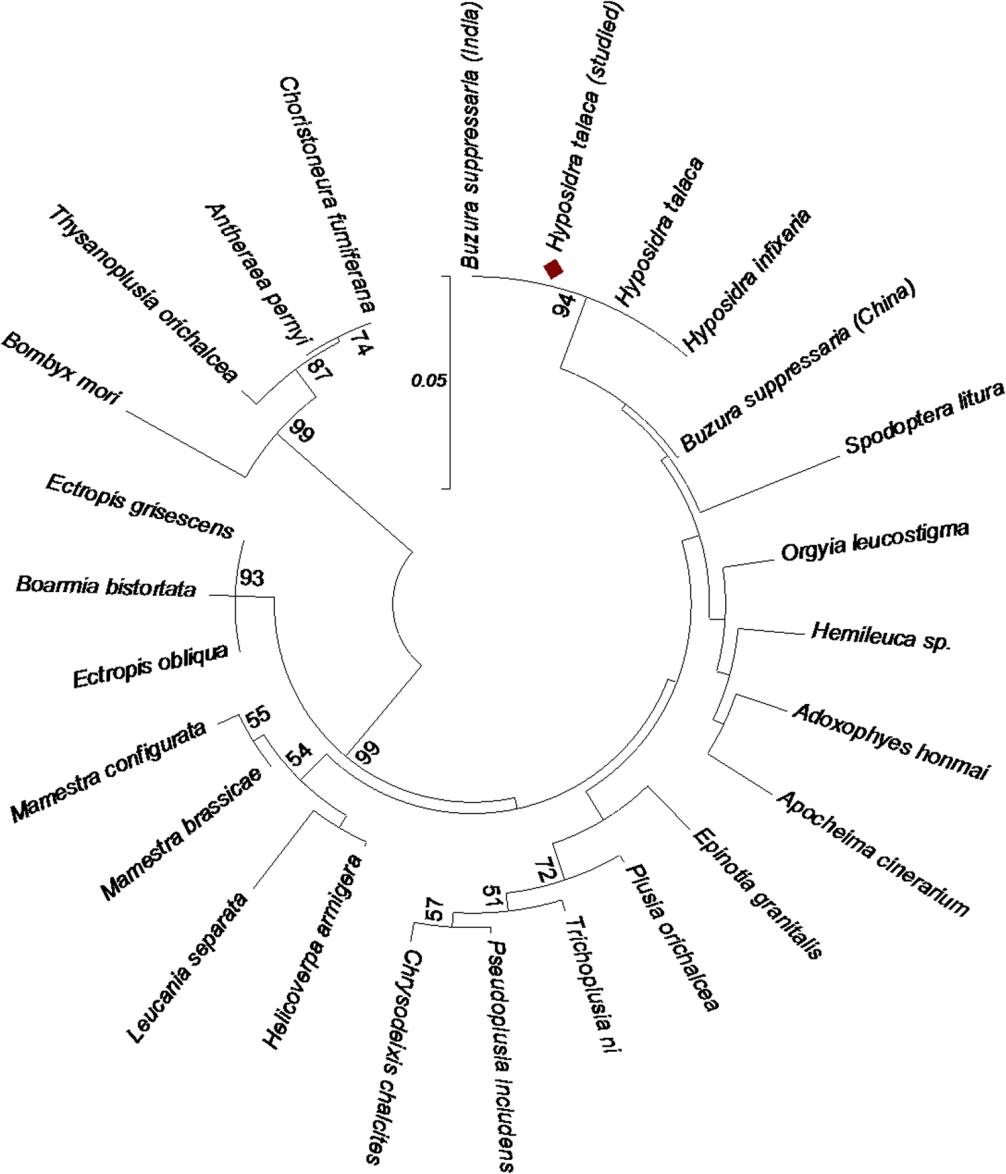
Phylogenetic analysis using the predicted amino acid residues from *Hyposidra talaca* NPV (HytaNPV-P) polyhedrin gene. The tree shows the relationship between HytaNPV-P and other NPVs. The tree was constructed by neighbor-joining algorithm using MEGA (v. 6.06). The branch numbers represent bootstrap probabilities of 1000 replicates. The *scale bar* represents 0.05 amino acid substitutions per site. The tree is rooted on the *Bombyx mori* NPV for comparison. Nodes with bootstrap value above 50% are shown. The analysis involved 26 NPV amino acid sequences; the position of HytaNPV-P polyhedrin is highlighted (in *red diamond*). All positions containing gaps and missing data were eliminated

Phylogenetic analysis of the polyhedrin gene was performed to find out the relationship between HytaNPV-P and other NPVs. The studied data were recognized in the NCBI taxonomy database as a HytaNPV which was referred here as HytaNPV-P. Phylogenetic analysis of the HytaNPV-P polyhedrin showed a high degree of relationship with a large number of published amino acid sequences. A neighbor-joining tree was generated from the amino acid sequences of 26 NPV polyhedrin genes. Phylogenetic analysis indicates that the NPV infecting the *H. talaca* species in the Terai region of India is closely related to HytaNPV, HyinNPV and BusuNPV of the Indian isolate and comes under the same clade (Fig. 5). This suggested that the collected virus-infected larvae from the Terai region were very likely infected by the same strain. It also appeared to have close relation with Chinese isolates of BusuNPV, supported by high bootstrap value. High protein sequence homology of the polyhedrin region of HytaNPV-P with BusuNPV (98%) of the Chinese isolate and phylogenetic analysis indicates that they are very closely related. Phylogenetic study clearly indicates that in India, a variant of Chinese isolate of BusuNPV infects the *H. talaca*.

### Bioassays

LC_50_ and LT_50_ values were represented in Table 2 for the tested isolates. The mortality ranged from 84 to 22% and it is clear that *H. talaca* larvae were susceptible to the applied concentrations. The result showed that the second instar was most susceptible to SMB isolate with an LC_50_ of 8.15 × 10^4^ POBs/ml. Larvae were also susceptible to infection with LC_50_ values of 3.87 × 10^5^, 1.89 × 10^5^ and 2.79 × 10^5^ POBs/ml, for RJB, WSB and TLP isolates, respectively, within the same time frame. The results of the study reflected the dose-dependent mortality, with the rising dose of POBs, the larval mortality showed an increasing trend. Inoculated larvae started to die at second day post-infection. In untreated control, no mortality was found. The estimated LT_50_ values varied from 3.25 to 6.67 days. An instance, for highest concentration (1 × 10^8^ POBs/ml) of the LT_50_ value of the WSB isolate was lowest at 3.25 days. When treated with the highest dose, the LT_50_ values were 3.5 days for the SMB isolate, 3.86 days for the RJB isolate and 3.89 days for the TLP isolate. On the other hand, the result also indicates a gradual increase in tolerance with lower concentration.

**Table 2:** Dose–mortality response of second instar *Hyposidra talaca* larvae on four geographic isolates of *H. talaca* nucleopolyhedrovirus (HytaNPV) within 7 days of inoculation

HytaNPV isolate	No. of tested 2nd instar larvae (*n*)	Concentration (POBs/ml)	HytaNPV caused mortality (%)	Slope ± SE	LC_50_ POBs/ml (95% confidence limits)	Chi square (*χ* ^2^)	LT_50_ (days)
SMB	90	1 × 10^8^	84	0.332 ± 0.035	8.15 × 10^4^ (3.39 × 10^4^–1.78 × 10^5^)	0.097	3.50
	90	1 × 10^7^	77				4.27
	90	1 × 10^6^	63				5.14
	90	1 × 10^5^	51				6.67
	90	1 × 10^4^	38				–
	90	1 × 10^3^	27				–
	90	Control	0				–
RJB	90	1 × 10^8^	77	0.297 ± 0.034	3.87 × 10^5^ (1.62 × 10^5^–9.44 × 10^5^)	0.108	3.86
	90	1 × 10^7^	67				4.80
	90	1 × 10^6^	53				5.80
	90	1 × 10^5^	43				–
	90	1 × 10^4^	32				–
	90	1 × 10^3^	22				–
	90	Control	0				–
WSB	90	1 × 10^8^	83	0.350 ± 0.035	1.89 × 10^5^ (8.73 × 10^4^–3.99 × 10^5^)	0.375	3.25
	90	1 × 10^7^	73				4.20
	90	1 × 10^6^	60				5.33
	90	1 × 10^5^	43				–
	90	1 × 10^4^	33				–
	90	1 × 10^3^	22				–
	90	Control	0				–
TLP	90	1 × 10^8^	78	0.300 ± 0.034	2.79 × 10^5^ (1.17 × 10^5^–6.65 × 10^5^)	0.081	3.89
	90	1 × 10^7^	69				4.75
	90	1 × 10^6^	56				5.86
	90	1 × 10^5^	45				–
	90	1 × 10^4^	33				–
	90	1 × 10^3^	23				–
	90	Control	0				–

Dose–mortality distribution with corresponding LC_50_ and LT_50_ values; Slope represents regression derivation. Data fit probit model by *χ* ^2^ test at *α* = 0.05, degree of freedom (*df*) = 5

## Discussion

Virus is now an important subject for the development of biopesticides and to maintain the ecosystem in the agricultural field. Search of a new natural isolate of virus, which contains better biopesticidal characteristics, to control insect pest is still an important aspect in biological control. In this regard, baculoviruses like NPVs are well known for their variability, frequent genetic variation and differences in their biology. These qualities remain intact even when the NPV isolates are sampled from the same species of different geographical locations (Cory et al. 2005).

*H. talaca* plays a leading role in reducing tea production throughout the north-eastern tea hub of India (Basu Majumdar and Ghosh 2004). *H. talaca* was reported as a tea defoliator in Java (Yunus and Ho 1980) and it was also reported as a major pest of mango, cacao and other forest trees (Entwistle 1972; Muniappan and Viraktamath 1986; Singh and Singh 2004). Some entomopathogenic bacteria and NPV, infecting the pest, have been reported from north-eastern part of India (Mukhopadhyay et al. 2010; Antony et al. 2011). However, commercial use of these microorganisms in this region is still far away. Unlike China and Japan, there is no considerable progress in using naturally occurring baculoviruses in integrated pest management of tea in India. In China, significant response of BusuNPV and EcobNPV against *B. suppressaria* and *E. oblique* made these NPVs commercially available (Hu et al. 1993; Ma et al. 2007). In India, HytaNPV can be an effective control measure against *H. talaca*. The result of the PCR study showed the presence of single NPV strain across the entire north-eastern tea hub of India. In lepidopteran NPVs, the full length of polyhedrin gene ranges from 483 to 747 bp (Jehle et al. 2006), and the 527 bp partial segment of the studied polyhedrin gene also fits within it. In the present study, the partial amplification of HytaNPV-P infecting *H. talaca,* showed 98% nucleotide sequence identity and 100% similarity in amino acids with previously published HytaNPV (Antony et al. 2011). HytaNPV-P also showed the same sequence identity with HyinNPV and BusuNPV (Indian isolate). This implies that HytaNPV-P is closely related to the aforesaid NPVs. Interestingly, HytaNPV-P also showed high sequence identity with the Chinese isolate of NPV infecting *B. suppressaria*. In this case, HytaNPV-P showed 98% similarity in amino acids with BusuNPV (acc. no. YP009001778, Chinese isolate) and only three amino acid differences were observed in the polyhedrin gene of HytaNPV-P and BusuNPV (Chinese isolate). This similarity indicates that HytaNPV-P is also related to the isolate of BusuNPV from China.

Phylogenetic studies have shown that HytaNPV-P, HytaNPV, HyinNPV and BusuNPV (Indian isolate) belong to same clade and the derived tree also encourages the fact that a variant of the Chinese isolate of BusuNPV (acc. no. YP009001778) infects the HytaNVP-P. However, full genome sequencing is necessary to confirm whether the HytaNPV-P altogether is a different virus or a different isolate of BusuNPV (China).

After ingestion of polyhedral occlusion bodies by larvae, NPV virions reach the midgut and spread to cause infection (Petrik et al. 2003). The results showed the effectiveness of the application of isolated NPV through tea leaves. In the present study, the SMB isolate of the Terai region showed higher LC_50_ value for second instar than the previously reported value of 2.8 × 10^3^ POBs/ml (from Terai region) by Mukhopadhyay et al. (2011). On the other hand, we found the RJB isolate (from Eastern Dooars), WSB isolate (from Western Dooars) and TLP isolate (from Central Dooars), which were respectively 5 times, 2.3 times and 3.4 times less active than the SMB isolate. The reasons for the variability may be the different geographic locations (Terai and Dooars) and differences in larval age (Payne et al. 1981) and feeding habit (Lacey et al. 2002). The number of virions per occlusion body and the host susceptibility differences to NPV (El-Salamouny et al. 2003) can be the other possible causes. However, for cross reference, there is no other report of bioassay available at this point for this particular tea pest. Stiles and Himmerich (1998) also observed higher LC_50_ of *Ac*MNPV against second instar *Heliothis zea* (i.e., 6.38 × 10^5^ POBs/ml). Second instar larvae of *Spodoptera littoralis* were also highly susceptible to the SpliMNPV at the higher concentration of 2 × 10^6^ POBs/ml, which caused a high rate of mortality than the lower concentration (Sutanto et al. 2014).

The virulence of baculoviruses is measured by the killing speed of the concerned virus to the insect host (Van-Beek and Hughes 1998) and is necessary to know the correct time for pest management programs. The highest inoculums of 10^8^ POBs/ml of HytaNPV showed rapid killing speed with LT_50_ value of 3.25 to 3.89 days after treatment for four isolates. Lower concentrations take longer time to meet the same level of larval mortality.

The result shows decreasing trend in mortality with the decreasing dosage. Although this relation between the rate of change in mortality with the lessening dosage is not directly proportional, as the dose decreases ten times the mortality rate is decreased well less than ten times. Previous studies also support this observation (Jankevica and Zarins 1999; Sajap et al. 2000; Sethuraman and Narayanan 2010; Mukhopadhyay et al. 2011; Sutanto et al. 2014). However, the regression result in Table 2 shows that a positive correlation exists between mortality and dosage, as all the slope values are positive and infinitesimal Chi squares signify that there is a good linear fit. Both the studies of LC_50_ and LT_50_ satisfied the insecticidal characteristics of HytaNPV. HytaNPV isolated from *H. talaca* is being developed as a biopesticide for the control of *H. talaca*.

## Conclusion

In this paper, we described the cloning and sequencing of a highly conserved region of polyhedrin gene and the insecticidal activity of HytaNPV. The PCR study revealed the presence of the same viral strain throughout the north-eastern tea belt. The high degree of phylogenetic agreement between the studied and previously reported HytaNPV polyhedrin gene sequence also supports this inference. This is the first report of sequencing of HytaNPV polyhedrin gene from the Terai region. The availability of HytaNPV may help in biopesticide production. The obtained results of bioassays support the use of HytaNPV as a potent biological control agent and a competent alternative to chemical insecticides in integrated pest management (IPM). The aforementioned characterization of the newly isolated HytaNPV would provide better understanding of the molecular properties of this virus and be helpful in the development of HytaNPV as a biopesticide.
